# Chemotherapy outcome predictive effectiveness by the Oncogramme: pilot trial on stage-IV colorectal cancer

**DOI:** 10.1186/s12967-016-0765-4

**Published:** 2016-01-12

**Authors:** Christophe Bounaix Morand du Puch, Michelle Nouaille, Stéphanie Giraud, Anaïs Labrunie, Sandrine Luce, Pierre-Marie Preux, François Labrousse, Alain Gainant, Nicole Tubiana-Mathieu, Valérie Le Brun-Ly, Denis Valleix, Angélique Guillaudeau, Laura Mesturoux, Béma Coulibaly, Christophe Lautrette, Muriel Mathonnet

**Affiliations:** Oncomedics SAS, ESTER technopole, 1 avenue d’Ester, 87069 Limoges, France; Centre d’Investigation Clinique, INSERM 1435, Centre hospitalier régional universitaire de Limoges Dupuytren, 2 avenue Martin Luther King, 87042 Limoges Cedex, France; Centre d’Épidémiologie, de Biostatistique et de Méthodologie de la Recherche, Centre hospitalier régional universitaire de Limoges Dupuytren, 2 rue du Dr Marcland, 87025 Limoges Cedex, France; Centre hospitalier régional universitaire de Limoges Dupuytren, service d’anatomopathologie, 2 avenue Martin Luther King, 87042 Limoges Cedex, France; Centre hospitalier régional universitaire de Limoges Dupuytren, service de chirurgie digestive, 2 rue du Dr Marcland, 87025 Limoges, France; Centre hospitalier régional universitaire de Limoges Dupuytren, service d’oncologie médicale, 2 avenue Martin Luther King, 87042 Limoges Cedex, France; Centre hospitalier régional universitaire de Limoges Dupuytren, service de chirurgie viscérale, 2 avenue Martin Luther King, 87042 Limoges Cedex, France; Centre hospitalier régional universitaire de Limoges Dupuytren, service de chirurgie digestive générale et endocrinienne, 2 avenue Martin Luther King, 87042 Limoges Cedex, France; Université de Limoges, Institut 145 GEIST, EA 3842 “Homéostasie cellulaire et pathologies”, Facultés de médecine et de pharmacie, 2 rue du Dr Marcland, 87025 Limoges Cedex, France

**Keywords:** Ex vivo model, Colorectal cancer, Oncogramme, Primary culture, Individualized tumor response testing, Chemosensitivity and resistance assay

## Abstract

**Background:**

Colorectal cancer (CRC) remains a major public concern. While conventional chemotherapeutic regimens have proved useful against advanced/metastatic diseases, progresses are to be made to effectively cure the large portion of patients not benefiting from these treatments. One direction to improve response rates is to develop chemosensitivity and resistance assays (CSRAs) efficiently assisting clinicians in treatment selection process, an already long preoccupation of oncologists and researchers. Several methods have been described to this day, none achieving yet sufficient reliability for recommended use in the clinical routine.

**Methods:**

We led a pilot study on 19 metastatic CRC patients evaluating capacity of the Oncogramme, a standardized process using tumor ex vivo models, to provide chemosensitivity profiles and predict clinical outcome of patients receiving standard CRC chemotherapeutics. Oncogramme responses were categorized according to the method of percentiles to assess sensitivity, specificity and concordance.

**Results:**

We report from a primary analysis a success rate of 97.4 %, a very good sensitivity (84.6 %), a below-average specificity (33.3 %), along with a global agreement of 63.6 % and a concordance between Oncogramme results and patients’ responses (Kappa coefficient) of 0.193. A supplementary analysis, focusing on CRC patients with no treatment switch over a longer time course, demonstrated improvement in specificity and concordance.

**Conclusions:**

Results establish feasibility and usefulness of the Oncogramme, prelude to a larger-scale trial. Advantages and drawbacks of the procedure are discussed, as well as the place of CSRAs within the future arsenal of methods available to clinicians to individualize treatments and improve patient prognosis.

*Trial registration*: ClinicalTrials.gov database, registration number: NCT02305368

**Electronic supplementary material:**

The online version of this article (doi:10.1186/s12967-016-0765-4) contains supplementary material, which is available to authorized users.

## Background

Colorectal cancer (CRC) is a major public health concern, being the third-most cancer diagnosed worldwide and the second-leading cause of cancer-related mortality in industrialized countries ([1]; GLOBOCAN data from the International Agency for Research on Cancer, available at http://globocan.iarc.fr/Pages/fact_sheets_cancer.aspx, accessed December 14, 2015), where combination of lifestyle and environmental factors are suspected to be responsible for such prevalence [2], besides heritable factors [3]. Moreover, as CRC declares at a median age of 68 in the US (National Cancer Institute data 2007–2011, available at http://seer.cancer.gov, accessed December 14, 2015), its occurrence is expected to continue rising in populations where life expectancy increases. Main treatment for early stage malignancy is surgery. Later stage diseases or patients in palliative care are treated with chemotherapy and/or targeted therapy in neoadjuvant/adjuvant settings, radiotherapy being additionally used for rectal cancer [3].

Several chemotherapeutic regimens are currently employed against advanced CRC. Most of them include: (1) the antimetabolite 5-fluorouracile (5-FU) or its precursors; (2) the thimidylate synthase inhibitor folinic acid (FA), enhancing the effects of 5-FU; (3) the topoisomerase inhibitor irinotecan; (4) the DNA-crosslinker oxaliplatin. 5-FU has been used for decades and still is a cornerstone for treatment of metastatic CRC, while irinotecan and oxaliplatin have been introduced for the last 15 years [4]. These molecules are used in combination doublet (5-FU and FA) or triplets (FOLFIRI: 5-FU, FA and irinotecan; FOLFOX: 5-FU, FA and oxaliplatin) for first- and second-line treatments, both triplets being equally effective [5]. These therapies are associated with known toxicities, whose severity depends on patients’ age or comorbidities and may worsen their overall condition, hence influencing therapeutic decisions.

A 2003–2009 US survey showed that metastatic CRC has a 5-year survival below 13 % [6]. However, advances in treatment have allowed significant amelioration in median overall survival (OS), now close to 24 months [7]. Such figures highlight the fact that prognosis can still be improved, either by developing more effective treatments, or better targeting existing therapies, or a combination of both.

Clinicians have access to complementary information to help them selecting a curative regimen, comprising pathological and molecular data as well as clinical characteristics of individuals. Yet, selection remains based on empirical decisions balancing therapeutic benefits and potential toxicity experienced by patients, and there is currently no efficient means of determining early the most appropriate chemotherapy. Inter- and intratumor heterogeneity, even within same histologic types [8], is mainly responsible for making responses to drugs highly unpredictable.

Because of such drawbacks, it appears that the “one-size-fits-all” approach is no longer suitable. In that context, a tool efficiently assisting clinicians in selecting drugs for a specific patient would be of high interest. This tool should provide data for improving response to treatments, i.e. ameliorate prognosis by suggesting better therapeutic options earlier in the disease, while avoiding multiple cycles of ineffective drugs with notable toxicity. Also, anticancer treatments and patient care being increasingly expensive [9], a complementary advantage would be reduction of the economic burden linked to overall care.

Presently, several approaches have been devised to achieve such goal. One direction is to identify relations between expression of specific genes/sets of genes and sensitivity or resistance to anticancer molecules [10]. Another direction is the use of chemosensitivity assays.

Chemosensitivity assays, generally termed individualized tumor response testing (ITRT) or chemotherapy sensitivity and resistance assays (CSRAs), have been developed for several decades [11], producing a dense literature that includes results from preclinical research, retrospective studies and assay-directed clinical trials on various types of cancer. Assay procedures look at different endpoints [12–19], which all share the common feature of being measured on ex vivo models, either whole/minced patient tissue samples, or primary cultures derived from these. In addition, most studies are directed toward advanced/metastatic/relapsed cancers, for which therapeutic options are limited, but also because such tumors provide larger quantities of material.

Usefulness and reliability of CSRAs have proven highly variable. The main reason put forward to explain this shortcoming is “the failure of such tests to identify clinically-active drugs” [20] and thus really impact patient survival. As a consequence, in its latest update of guidelines regarding use of CSRAs, the American Society of Clinical Oncology (ASCO) still does not recommend such tests outside of the clinical trial setting, but maintains as a priority their continued evaluation because of their potential importance [21]. Nevertheless, chemosensitivity assays are already commercially available in North America [22–24], Japan [25] and United Kingdom [26].

The company Oncomedics has developed several ex vivo primary culture cancer models. They are obtained thanks to the use of chemically-defined media, which allow tissue preservation, dissociation and subsequent culture, while maintaining intrinsic heterogeneity of original tumor cell subpopulations. Hence, when transferred to an in-house fully-standardized methodology termed “the Oncogramme”, such models have appeared suitable to determine response profiles to chemotherapeutic agents on CRC [27], breast [28] and ovarian [29] cancers, enrich CRC cell lines in immature cells [30], and even predict sensitivity of breast cancer to targeted treatments [31].

Because of the demonstrated relevance of these models, we decided to complete a prospective pilot clinical trial aiming at evaluating (1) technical feasibility of the Oncogramme in a clinical context, and more importantly (2) its predictive effectiveness for a small cohort of stage-IV CRC patients receiving currently approved chemotherapies as part of their treatment protocol. Beyond an excellent success rate for effective patient profiling, we report from a 19-patient cohort a very good sensitivity but a below-average specificity, weakening concordance but still allowing a global agreement of 63.6 % (percentage of patients whose response to drugs was correctly predicted by the Oncogramme). Supplementary analysis, focusing on a subset of patients having received only one chemotherapeutic treatment for a longer time course, displayed improved specificity, agreement and concordance. These results overall demonstrate practicability and usefulness of the Oncogramme, and indicate future directions for global enhancement of the method.

## Methods

### Patient selection

Only stage-IV colorectal cancer patients were recruited, because of feasibility of metastatic lesions follow-up. Criteria for inclusion and exclusion of patients are presented in Additional file 1: Table S1.

### Sample selection

Fresh stage-IV colorectal cancer specimens were anonymously obtained from non-objecting patients treated at the *Centre Hospitalier Régional Universitaire* (CHRU) Dupuytren (Limoges, France) from January 2011 till December 2012, and set to undergo primary tumor resection. Scientific and clinical significance of the study was validated by the *Délégation à la Recherche Clinique et à l’Innovation* (DRCI). Study protocol and case report form were approved by the *Comité de Protection des Personnes* (CPP) Sud-Ouest et Outre-Mer IV. Authorizations were obtained from the *Comité Consultatif sur le Traitement de l’Information en Matière de Recherche dans le domaine de la Santé* (CCTIRS) and the *Commission Nationale Informatique et Libertés* (CNIL).

Following surgical resection, primary lesions were histologically qualified by a pathologist through systematic analysis of sections facing site of sampling. If tumor was large enough to provide tissue for both diagnosis and Oncogramme purposes, a non-peripheral yet non-necrotic portion (100–200 mm^3^) of each fresh, unfixed tissue was collected in OncoMiD-Via for colon conservation medium (Oncomedics) within 2 h of resection and stored at 4 °C for a maximum of 48 h. Site of invasion of colon/rectum wall was carefully preserved for diagnosis, and staging was determined according to TNM 7th edition staging system [32]. Remote lesions had to be measurable according to response evaluation criteria in solid tumors (RECIST 1.1, described in [33]), and their evolution following treatment was also assessed based on these criteria. Initial pre-surgery identification of metastatic lesions was performed thanks to computed tomography (CT), magnetic resonance imaging (MRI), and/or 18F-fluorodeoxyglucose positron emission tomography-computed tomography (FDG-PET-CT). Per-surgery observations completed identification in patients for whom metastases had not been previously discovered.

### Sample processing and primary culture

Samples reserved in OncoMiD-Via for colon were transported according to UN3373 classification standards. Dissociation was performed with OncoMiD-Diss for colon dissociation kit (Oncomedics), involving mechanical and chemical steps [27]. Cell viability was assessed by trypan blue exclusion assay (Sigma Aldrich). Cells were seeded at a density of 4–8.10^5^ cells/mL in OncoMiD for colon serum-free, defined medium (Oncomedics), supplemented with 2.5 µg/mL amphotericin B (Sigma Aldrich) in EasyFlask, polystyrene Nunclon-treated culture dishes with filter caps (Nunc). Cultures were kept at 37 °C in a humidified incubator (Binder CS 150) in a 95 % air 5 % CO_2_ atmosphere. Medium containing amphotericin B was renewed after 5 days.

### Chemotherapies

Stock solution of chemotherapies (all purchased from Sigma Aldrich) were prepared as follows: 5-fluorouracile (5-FU) was diluted at 1 mg/mL in phosphate-buffered saline (PBS) 10 % dimethylsulfoxyde, while folinic acid (FA), irinotecan and oxaliplatin were diluted at 5 mg/mL in H_2_O.

### Exposure to chemotherapies

After 7 days of culture, cells were collected and centrifuged for 10 min at 300 g, their viability was assessed with trypan blue, and 8-well lab-tek culture chambers (Nunc) were seeded. Each well received 5.10^4^ live cells in final volume of 500 µL OncoMiD for colon. For one patient, a complete experiment included 4 conditions in monoplicate: untreated; 5-FU and FA; FOLFIRI; FOLFOX. Chemotherapies were added at previously determined final concentrations [27]: 5-FU = 25; FA = 5; irinotecan = 100; oxaliplatin = 150 µg/mL. Culture chamber was placed back in incubator for 72 h.

### Cell viability/mortality labeling

Following exposure to treatments, cell viability was assessed through a fluorescent triple labeling. Briefly, cells were incubated for 45 min in PBS containing 4 µM acetomethoxy derivate of calcein and 0.1 µM ethidium homodimer-1 (LIVE/DEAD^®^ Viability/Cytotoxicity kit, Life Technologies). They were then washed with PBS and fixed in PBS 4 % formaldehyde (Sigma Aldrich) for 10 min. After a wash with PBS, total cell population was labeled through incubation in H_2_O containing 0.5 µg/mL 4′,6-diamidino-2-phenylindole (DAPI; Sigma Aldrich). Cells were then washed 3 times in PBS, once in H_2_O, and dried. Finally, slides were mounted with glycerol/gelatin mounting medium (Sigma Aldrich) and stored at −20 °C until readout.

### Cytotoxicity analysis

Cells were observed with a fluorescence microscope (Nikon). Multi-channel pictures randomly covering the surface of each well were taken using NIS-Elements BR 3.1 software (Nikon). Variable number of pictures were taken for each patient, to provide sufficient number of cells (at least 1000) for accurate results. Live, dead and overall cell populations were counted and percentage of dead cells was determined for each condition. Finally, for each patient, ratios of death percentages for treated cells to death percentages for untreated cells were computed. Whole endpoint analysis was solely performed by one person (CBMP). Results were not communicated to clinicians.

### Results categorization and statistical analysis

After each clinical evaluation (variable time interval, usually 2–4 months), patients were categorized into responders (complete or partial response, stable disease) and non-responders (progressive disease) to treatments according to RECIST 1.1. Results were not communicated to Oncogramme reader until end of study. Oncogramme results were categorized according to the method of percentiles [34]: patients highly sensitive to treatments were those for which ratios were above 75th percentile; intermediate sensitive patients included patients for which ratios were between 25th and 75th percentiles; resistant patients included patients for which ratios were below 25th percentile. After data verification, database was frozen and statistical analysis was computed. All quantitative variables were described by mean ± standard deviation, minimum, maximum, median and interquartile range. Qualitative variables were described by frequencies and percentages. Capacity for the Oncogramme to identify responders as sensitive ex vivo to the chemotherapy they actually received was defined as sensitivity. Capacity for the Oncogramme to identify non-responders as resistant ex vivo to the chemotherapy they actually received was defined as specificity. These measures of validity were estimated using a contingency table crossing results observed on patients and Oncogramme results. Their 95 % confidence intervals (CIs) were calculated with the exact method. To quantify concordance between Oncogramme results following categorization and results observed on patients, Kappa coefficient, ranging from −1 to 1, was also estimated with a 95 % confidence interval. For its interpretation, categories from Landis and Koch [35] were used.

### Quality control

Reporting of clinical data, deviations from initial protocol, assay results and overall writing of manuscript followed STAndards for the Reporting of Diagnostic accuracy studies (STARD, http://www.stard-statement.org/).

## Results

**Fig. 1:**
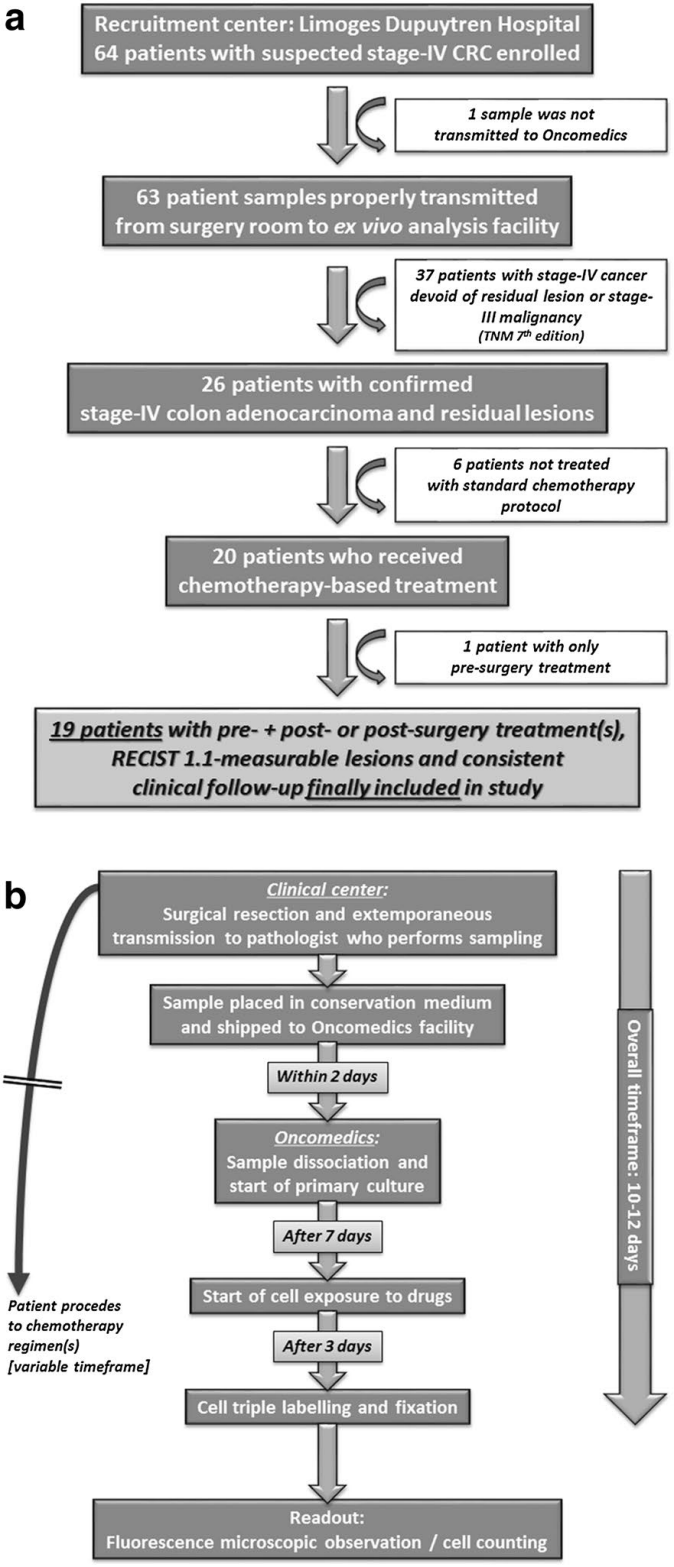
**a** Overview of patient selection process in the pilot trial, from initial recruitment to final inclusion. From the 64 individuals originally recruited, exclusion and inclusion criteria allowed to finally select 19 patients with stage-IV CRC, pre- + post- or post-surgery treatment, RECIST 1.1-measurable lesions and consistent clinical follow-up. **b** Overview of the Oncogramme experimental procedure, from surgery to readout. Viable samples were recovered and processed to obtain primary cultures that were subsequently utilized for realization of the Oncogramme by exposure to chemotherapeutic drugs and cell death analysis. Whole time course was inferior to 2 weeks

Diagram presenting the inclusion process appears in Fig. 1a. Initially, 64 patients were enrolled, with 63 samples properly transmitted from surgery room to Oncomedics’ facility. Stage-IV colon carcinoma was diagnosed for 26 patients, 6 of which did not actually receive any chemotherapy. Finally, 1 patient received pre-surgery chemotherapy only, while 19 patients received either pre- and/or per- and post- (3 patients) or only post-surgery (16 patients) chemotherapy. These 19 patients were finally included in the study.

Flowchart summarizing the whole Oncogramme procedure is presented in Fig. 1b. The only deviation from protocol occurred for 2 patients (n° 12 and 18), whose samples were processed 3 days after surgery instead of the 2 days initially planned because of improper transmission of information between pathology laboratory and Oncomedics. Nevertheless, it did not impair completion of procedure, quality of cultures, and gathering of results for these individuals.

Table 1 presents main characteristics of the 19 included patients. Median age was 69 with an interquartile range of (62; 79) (mean = 65, range = 37–82), and an almost equal repartition of individuals according to sex (9 males, 10 females) was observed. Body mass index (BMI) showed the majority (52.6 %) of patients presented a normal range weight, while 10.5 % were considered underweight (BMI < 18.5), 21.1 % overweight (25 < BMI < 30), and 15.8 % obese (BMI > 30). American Society of Anesthesiologists (ASA) physical status score was used to assess fitness of patients before surgery: it indicated a severe systemic disease for 42.1 % patients (score = 3). Both indexes suggested potential preclusion of certain chemotherapeutic regimens for patients at risk. Localization of primitive lesions spanned all colon segments (left/descending colon: 42.1 %; sigmoid: 21 %; right/ascending colon: 31.6 %) and rectum (5.3 %). Metastases were identified pre-surgery in 57.9 % cases, while per-surgery observations completed identification of remote lesions.

**Table 1:** Main characteristics of advanced CRC patients included in the pilot trial and their malignancies

Subject ID	Patient characteristics				Disease characteristics					
					Localisation of primary lesion	Pre-surgery identification of metastases				Per-surgery identification of metastases
	Age (years)	Sex	Body mass index (BMI)	Physical status score (ASA)		CT- visible metastases	MRI-visible metastases	FDG PET scan- visible metastases	Localisation of per-surgery identified metastases	
01	63	M	23.46	2	Left/descending colon	Yes	N/A	N/A	Abdomen + peritoneal carcinomatosis	N/A
02	37	F	35.03	2	Left/descending colon	No	N/A	N/A	–	Yes
03	79	F	16.11	2	Left/descending colon	Yes	Yes	N/A	Pelvic lymph nodes	N/A
04	63	F	24.65	2	Right/ascending colon	Yes	N/A	N/A	Liver	N/A
05	45	F	21.50	3	Sigmoid	Yes	N/A	N/A	Ovary + peritoneal carcinomatosis	N/A
06	39	F	19.20	3	Sigmoid	Yes	Yes	Yes	Liver, lungs	N/A
07	82	F	21.09	3	Right/ascending colon	No	N/A	N/A	–	Yes
08	62	M	31.25	3	Sigmoid	N/A	N/A	N/A	–	Yes
09	79	M	24.80	2	Right/ascending colon	Yes	N/A	N/A	Liver	N/A
10	70	M	22.57	2	Left/descending colon	No	N/A	N/A	–	Yes
11	80	F	21.23	3	Rectum	Yes	N/A	N/A	Liver	N/A
12	69	M	26.89	3	Right/ascending colon	Yes	N/A	N/A	Liver, lungs	N/A
13	80	F	22.19	2	Right/ascending colon	No	N/A	N/A	–	Yes
14	81	M	30.07	2	Right/ascending colon	No	N/A	No	–	Yes
15	73	M	24.49	3	Left/descending colon	Yes	N/A	N/A	Liver, lungs	N/A
16	62	M	28.41	1	Left/descending colon	No	N/A	N/A	–	Yes
17	65	F	38.57	2	Sigmoid	Yes	N/A	N/A	Peritoneal carcinomatosis	N/A
18	39	F	17.36	2	Left/descending colon	Yes	N/A	N/A	Liver, lungs	N/A
19	75	M	29.39	3	Left/descending colon	No	N/A	N/A	–	Yes

Table 2 presents chemotherapy regimens followed by all patients, as well as their responses to treatment after each evaluation. For first cures, 31.6 % patients received 5-FU with or without FA, 10.5 % received FOLFIRI, and 57.9 % received FOLFOX. Twenty-one percent patients received consecutive lines of treatments involving 2 or 3 chemotherapies. Twenty-one percent patients died before the end of study, all of them because of disease progression. Minimal follow-up time was 13 months for patients who did not die before study completion. Patients were subsequently categorized into responders and non-responders. It is noteworthy that 8 patients were administered angiogenesis inhibitor bevacizumab during the course of their treatment, six of which as soon as first cure. Since our ex vivo two-dimensional model is devoid of microenvironment and vascular network, this antibody could not be tested through such configuration of the Oncogramme. However, as an anti-angiogenic agent, it was shown not to have an effect on response rates and survival by itself, but rather to reinforce the action of chemotherapies [36]: this is why we decided to include patients that received this molecule. Also, 1 wild-type *KRAS* patient received panitumumab, which targets EGF receptor to inhibit cell proliferation. In our study, administration of this antibody was non-concomitant with any chemotherapy, thereby not interfering with comparison of patient outcome following a 5-FU first line.

**Table 2:** Treatments and clinical responses of advanced CRC patients included in the pilot trial

Subject ID	First cure Results of first evaluation	Second cure Results of second evaluation	Third cure Results of third evaluation	Survival time (months) at end of study
01^a^	5-FU + Radiotherapy Disease progression	8 C FOLFIRI Disease progression	1 C 5-FU Disease progression	27
02	4 C FOLFOX Stable disease	4 C FOLFOX Stable disease	4 C FOLFOX Complete response	31
03^a^	1 C 5-FU Disease progression	4 C Panitumumab Disease progression	– Disease progression	08
04^a^	4 C FOLFOX + Bevacizumab Partial response	4 C FOLFOX + Bevacizumab Stable disease	4 C Bevacizumab Disease progression	21
05^b^	6 C FOLFOX + Bevacizumab Stable disease	4 C FOLFOX + Bevacizumab Disease progression	Bevacizumab Disease progression	25
06^b^	4 C FOLFIRI + Bevacizumab Stable disease	4 C FOLFIRI + Bevacizumab Stable disease	4 C Bevacizumab Disease progression	24
07	3 C 5-FU Stable disease	4 C 5-FU Stable disease	1 C 5-FU Complete response	24
08	3 C FOLFOX Stable disease	4 C FOLFOX Disease progression	4 FOLFIRI Disease progression	31
09^a,b^	4 C FOLFOX Disease progression	4 C FOLFIRI + Bevacizumab Stable disease	4 C FOLFIRI Stable disease	14
10	8 C FOLFOX Stable disease	4 C FOLFOX Stable disease	N/A Stable disease	17
11	3 C 5-FU Stable disease	N/A Stable disease	N/A Stable disease	16
12	4 C FOLFIRI + Bevacizumab Stable disease	4 C FOLFIRI + Bevacizumab Partial response	4 C Bevacizumab Stable disease	15
13	3 C 5-FU Stable disease	2 C 5-FU Stable disease	2 C 5-FU Stable disease	15
14	8 C FOLFOX Stable disease	4 C 5-FU + Folinic acid Stable disease	N/A Stable disease	15
15	4 C FOLFOX + Bevacizumab Partial response	9 C FOLFOX + Bevacizumab Stable disease	N/A Partial response	14
16	6 C FOLFOX Stable disease	4 C FOLFOX Stable disease	4 C FOLFOX Stable disease	14
17	7 C FOLFOX + Bevacizumab Disease progression	5 C FOLFOX + Bevacizumab Stable disease	N/A Stable disease	14
18	4 C FOLFOX Partial response	5 C FOLFOX + Bevacizumab Partial response	N/A Partial response	14
19	3 C 5-FU Stable disease	3 C 5-FU Stable disease	3 C 5-FU Disease progression	13

Chemotherapy regimens received by each patient during the course of their treatment, and ensuing clinical outcome (disease progression, stabilization, partial or complete response) determined through three consecutive evaluations. Survival time at completion of study is also provided

^a^Patient died before end of study as a result of CRC progression

^b^Received pre- and or per-surgery chemotherapy

Contamination-free primary cultures were obtained in 100 % cases. Only sample of patient 06 did not provide enough cells to test all 4 planned experimental conditions: in addition to untreated well, cells were only exposed to 5-FU and FA, while the patient actually received FOLFIRI. Hence, a success rate of 74/76 experimental conditions (97.4 %) was obtained. Comparison between assay results and clinical outcome was possible for 18/19 patients (94.7 %) and, since 4 patients received two or more lines of different treatments, we were finally able to compare clinical outcome with ex vivo assay results in 22 cases.

Mortality of untreated cells after 10 days of culture was extremely variable from patient to patient (range 8.2–30.9 % dead cells; median: 18.1 %). In order to better compare results among subjects, we chose to report chemotherapy responses normalized to references obtained on untreated cells. Ranges of ratio for each chemotherapeutic condition are presented in Table 3. As results were not normally distributed, median was used. Twenty-fifth and 75th percentiles were computed. Because of the use of monoplicates, no coefficient of variation was determined.

**Table 3:** Main Oncogramme results following determination of cytotoxicity on individual primary cultures

Treatment	Median	Minimum	Maximum	25th percentile	75th percentile
5-FU + FA	1.343	0.895	2.102	1.029	1.701
FOLFIRI	1.633	0.788	2.883	1.186	1.965
FOLFOX	1.787	1.147	3.613	1.579	2.090

Figures presented are derived from ratios (% dead cells for treated condition/ % dead cells for untreated condition) obtained for the 19 advanced CRC patients included in the pilot trial. Mean and median values, as well as standard deviation and ranges are provided, as well as 25th and 75th percentiles

Table 4 presents ex vivo results for each patient following their categorization according to the method of percentiles. Highly and intermediate sensitive patients were gathered in the “sensitive” category. Table 5 matches clinical responses with Oncogramme profiles. This table allowed determining Oncogramme sensitivity at 84.6 % [11/13, 95 % confidence interval (CI) (54.5; 98.1)]. Consecutively, specificity was determined at 33.3 % [3/9, 95 % CI (7.5; 70.1)]. Oncogramme results were in accordance with patient outcome in 14/22 (63.6 %) cases. Kappa coefficient was measured at 0.193 [95 % CI (−0.196; 0.582)], indicating a real concordance between ex vivo and clinical results of 19.3 % non-attributable to randomness (very weak).

**Table 4:** Oncogramme results for the 19 advanced CRC patients included in the pilot trial

Subject ID	Treatment		
	5-FU + FA	FOLFIRI	FOLFOX
01	S	S	S
02^a^	S	S	I
03	S	S	S
04^a^	R	I	I
05^a^	I	R	R
06	I	N/D	N/D
07^a^	S	I	I
08^a^	I	I	I
09	R	S	S
10^a^	I	R	S
11	I	R	I
12^a^	I	I	I
13^a^	S	I	I
14	I	I	S
15^a^	I	R	R
16^a^	I	R	R
17^a^	I	I	R
18^a^	R	S	I
19^a^	R	I	I

Results were categorized according to percentile thresholds (R = resistant < 25th percentile < I = intermediate sensitive < 75th percentile < S = sensitive; N/D = not determined). Oncogramme results for treatments that were actually given to patients are underlined

^a^Indicates the 13 patients selected for the supplementary analysis, which were those who received equivalent chemotherapeutic treatments over the course of at least two evaluations

**Table 5:**
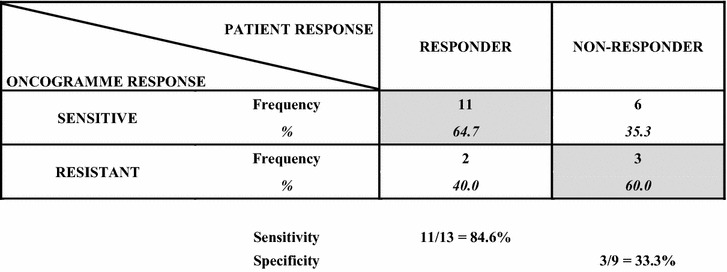
Correlation table matching patient responses with results of the Oncogramme assay (principal analysis)

Advanced CRC patient responses (responder or non-responder to treatment) were correlated with results of the Oncogramme assay (sensitive or resistant) to identify true positives (patients termed as sensitive and that actually responded to treatment) and true negatives (patients termed as resistant and that actually did not respond to treatment). True positives and true negatives are highlighted in grey. Sensitivity (percentage of true positives) and specificity (percentage of true negatives) are also given

To investigate predictive capacities of the Oncogramme on longer course treatments and on tumors whose sensitivity profile to other chemotherapies could not be altered by a first line regimen, a supplementary analysis was performed only on those patients who received at least first and second cures identical in their chemotherapy composition (no treatment switch after first evaluation). Thirteen patients were isolated, whose responses to treatments were compared to Oncogramme results (Table 4, ''a'' labelled patients). For that subgroup, sensitivity was 70.0 % [7/10, 95 % CI (34.75; 93.33)], specificity was 66.7 % [2/3, 95 % CI (9.43; 99.16)], and agreement between test results and patient outcome was visible in 9/13 (69.2 %) cases. Kappa coefficient was measured at 0.2973 [95 % CI (−0.2184; 0.8130)], indicating a real concordance between ex vivo and clinical results of 29.7 % non-attributable to randomness (weak), an improvement from the primary analysis. Additionally, identical sensitivity and specificity were obtained when 25th and 75th percentiles were re-computed using results obtained for these sole 13 patients, and a similar conclusion was drawn for real concordance (data not shown).

Additional file 2: Figure S1 presents examples of Oncogramme profiles for 4 patients, illustrating the heterogeneity of responses from patient to patient and from drug to drug.

## Discussion

Because of lack of recommendation from authorities, there is currently no gold-standard for CSRAs. Also, as methods largely differ in ex vivo models (histoculture, two-dimensional primary cultures, spheroids) as well as endpoints, it is difficult to closely compare them [37–42]. Nevertheless, our STARD-described pilot study demonstrated technical advantages for the Oncogramme, owing to its original design and full standardization. First, necessary amount of specimen was small enough so that both complete diagnosis and Oncogramme procedure were possible. Then, use of proper decontamination procedure resulted in contamination-free primary cultures for all cases. Contaminations are a notable hurdle in CSRAs, especially those involving CRC samples. They may account for a failure rate >10 % [40], which is not acceptable because of clinical importance of patients’ samples.

It is noteworthy that most procedures previously described were performed on fragments or cells that either did not undergo a primary culture step or, when cultured, were placed in serum-containing media. Process for the Oncogramme includes a non-passaged two-dimensional primary culture step. Downsides of primary culture include difficulties to avoid fibroblast contamination, loss of tumor architecture and cell–cell interactions, the two latter potentially being critical elements [38, 41]. Also, culture medium appears to be a decisive factor, since it must be able to preserve heterogeneity of tumor characteristics while allowing cell analysis through an easily manageable assay. To counter these disadvantages, we have designed defined medium OncoMiD for colon, providing a permissive environment for tumor cells while compromising survival of fibroblasts [27]. Overall, our primary culture conditions are appropriate for: (1) favoring tumor cell maintenance; (2) avoiding clonal expansion of rapidly dividing tumor cells, thus preserving sample heterogeneity; (3) eliminating unwanted cell subpopulations. Use of this medium resulted in 100 % success in primary culture, and comparison between patient response and assay results was possible in all but one case, a higher figure than previous reports [40, 41]. Ensuing increase in timeframe is not detrimental to patients, since first-line chemotherapy regimen is usually not started before several days or weeks after surgery [43].

One of the main drawbacks of several approaches is their capacity to detect only actively proliferating cells, while not appraising programmed cell death consecutive to drug treatment [20]. This is notably the case for clonogenic assays, which display moderate sensitivity [44]. Such tests usually fail at predicting clinical outcome. Our endpoint, indistinguishably assessing both metabolic capacity and membrane integrity, allows targeting all cells within the primary culture, regardless of proliferative/quiescent state and death pathway. Potentially dormant cell subpopulations (reversibly non-dividing, in G_0_ phase) are hence visible. Also, MTT-based assays tend to lack sensitivity because of their optical density-based endpoint. The Oncogramme, relying on direct cell count, displays higher sensitivity, though currently at the expense of a longer analysis time than plate readers. This issue should be worked out for future studies. Also, absence of replicates prevented us to improve level of confidence of results: this will be reinforced in next studies by scaling down the protocol to allow performing replicates but also working on smaller samples.

CSRAs such as the Oncogramme aim at improving clinical response to first-line treatments, since any failing line decreases chances of effective cure. Average response rates observed in studies involving more than 100 metastatic CRC patients and published in the last 15 years, for the 3 regimens employed here or their analogous, utilized as first-lines, were recently gathered [45]. Compiled figures are: 5-FU and folinic acid = 21.8 % ± 7.1 responders; 5-FU and folinic acid and irinotecan = 40.8 % ± 10.7; 5-FU and folinic acid and oxaliplatin = 46.4 % ± 7.7. This confirms a large portion of CRC patients empirically treated with current standards-of-care ultimately do not respond to administered therapies. Prospective studies also evaluated the predictive capacity of CSRAs, but recent reviews compiling up-to-date results for several pathologies are lacking [46]. Besides feasibility of the overall procedure in the clinical setting, primary goal of our pilot study was to determine whether the Oncogramme was capable of predicting objective response of stage-IV CRC patients to drugs currently in use. Despite a very low concordance (0.193), at least partly due to small size of the cohort, we achieved through the principal analysis a very good sensitivity [84.6 %, 95 % CI (54.5; 98.1)], demonstrating a propensity to identify patients sensitive to drugs or combinations (responders). On the other side, non-responding patients represented 60 % (3/5) of negative assay results. However, below-average specificity [33.3 %, 95 % CI (7.5; 70.1)] was obtained, meaning rate of false-positives was superior to that of true-negatives. When selecting for a supplementary analysis patients that received only a single type of regimen over the course of first, second and sometimes third cures, the Oncogramme was able to detect responders in 70 % cases and non-responders in 66.7 % cases. Despite being obtained on a low number of patients (n = 13), these figures suggest the test may be effective at predicting a patient response to a specific treatment (1) on a longer time-course (at least two evaluations); (2) when we avoid comparison between ex vivo chemosensitivity of naive tumor cells and in situ responses of primary and/or distant lesions potentially affected by previous rounds of chemotherapies, as mechanisms of acquired cross-resistances are not fully understood yet [47]. Our approach still allowed an acceptable sensitivity while reducing the risk of using drugs that will not be efficient. These predictivity indicators clearly need to be reinforced on a larger cohort, where inclusion criteria will be adapted so as to select patients that received a particular treatment for a sufficiently long period of time to more precisely define the resistance/sensitivity limit.

As previously pointed out, the Oncogramme and all other CSRAs do not distinguish between cell subpopulations making up the tumor ex vivo model. Responses provided by these tests are based upon global behavior of all cells when exposed to drugs: a generally responsive tumor tissue can thus translate into a test result categorizing it as “sensitive”, while cancer stem cells, thought to be responsible for neoplastic resurgence and resistance to further treatment [48], will not be identified. This might explain the high patient death rate associated with false-positives in our principal analysis: indeed, among patients with false-positive Oncogramme responses (individuals 01, 03, 08 and 09), 3 eventually died of their disease. In addition, it is important to notice that in vitro/ex vivo responses are generally more exacerbated than in vivo responses. This explains why numerous published works are actually more accurate at predicting resistance than sensitivity to drugs [38, 41], a downside that has attracted criticism from the ASCO. Compared to whole organisms [20], and despite their relevance [49], in vitro/ex vivo systems lack surrounding tissues and microenvironment that regulate drug delivery and tumor/cell behavior and ultimately modify patient response. Tumor ex vivo reconstruction by assembling its components in co-culture systems may help overcome this hurdle, but such solution appears difficult to apply to the clinical setting. Development of a more relevant model, thanks to adequate sample processing and culture conditions applicable to routine use, such as those included in the Oncogramme procedure, would be an equally elegant solution. Another important issue that should be considered for future developments of all CSRAs regards the representativeness of the working samples: they should encompass all characteristics of a patient’s own pathology, since variable cell death rates may be observed in superficial and deep parts of CRC tissue [39] while primary CRC tumor makeup, and thereby chemoresponses, may significantly differ from that of distant metastases [50].

Molecular approaches measuring the expression of markers potentially predictive of response to drugs are also widely considered for personalized medicine [51]. Up to now, however, CSRAs have proved to perform better in predicting clinical response to treatments in direct comparison studies [42, 52]. To maximize response rates, but also to understand mechanisms underlying intrinsic resistances and neoplastic resurgence, a synergistic framework combining CSRAs with relevant gene status studies could be envisioned [10, 47]. In addition, the Oncogramme appears suitable for evaluation of targeted therapies [31] as well as experimental molecules, cross-resistance drugs and synergistic/additive effects. Only in such context of accumulated evidence will the Oncogramme and other CSRAs best support clinicians in their decision process, increasing drugs’ therapeutic index and improving patients’ quality of life. A recent observational study showed physicians are actually willing to use results of CSRAs when available, and adapt their treatment protocol accordingly [53]: this establishes that potential role of such assistance and its diffusion through the medical community are not negligible.

Clinical feasibility of the fully-standardized Oncogramme was demonstrated on more than 60 patients. Despite a still weak concordance, mostly due to a low specificity that should be improved through more stringent patient selection criteria, a good agreement with clinical observations was reached. Particularly, the very good sensitivity shows that the Oncogramme profiles may be employed by clinicians with a positive-only outcome. It is now necessary to strengthen these preliminary results through a larger scale, randomized multicentric prospective trial that will compare performances of Oncogramme-directed treatments and empirical, physician-directed treatments on CRC tumors. Such study will also help adjusting sensitive/resistant limits for each chemotherapy or combinations.

## Conclusions

The goal of CSRAs is to assist clinicians in selecting the most appropriate treatment for a cancer patient by providing additional data regarding the chemosensitivity/-resistance capacities of a her/his tumor. The fully-standardized method of the Oncogramme, applied to a small cohort of metastatic CRC patients, was able to identify with an excellent success rate and a very good sensitivity those who respond to conventional chemotherapeutic treatments. Specificity was below average, denoting a weakness of the method at pointing out resistances. However, we also showed that more stringent selection criteria (longer follow-up of patients with no treatment switch) may help to drastically enhance this latter indicator, thereby ameliorating the global method efficiency. Despite the fact that our data need to be strengthened through a larger study, improvement of clinical response rates for standards-of-care appears possible through the Oncogramme.

## Additional files

10.1186/s12967-016-0765-4 Criteria for inclusion and exclusion of CRC patients used in the Oncogramme pilot trial.
10.1186/s12967-016-0765-4 Oncogramme profiles for 4 metastatic CRC patients included in the study. These profiles illustrate the heterogeneity of responses that occur from patient to patient, and for the three administered therapies. Bold dotted vertical line indicates on each graph the positivity threshold: an Oncogramme result indicative of resistance to the considered treatment is materialized by a red column extending to the left of threshold, an Oncogramme result indicative of sensitivity is materialized by a blue column extending to the right of threshold.

## Abbreviations

CCTIRS: *Comité Consultatif sur le Traitement de l’Information en matière de Recherche dans le domaine de la Santé*
CHRU: *Centre Hospitalier Régional Universitaire*
CI: confidence interval
CNIL: *Commission Nationale Informatique et Libertés*
CPP: *Comité de Protection des Personnes*
CRC: colorectal cancer
CSRA: chemosensitivity and resistance assay
CT: computed tomography
DAPI: 4′,6-diamidino-2-phenylindole
DRCI: *Délégation à la Recherche Clinique et à l’Innovation*
FA: folinic acid
FDG-PET-CT: 18F-fluorodeoxyglucose positron emission tomography-computed tomography
5-FU: 5-fluorouracile
ITRT: individualized tumor response testing
MRI: magnetic resonance imaging
PBS: phosphate-buffered saline
RECIST: response evaluation criteria in solid tumors
