# Supplementary material for: Chemotherapy outcome predictive effectiveness by the Oncogramme: pilot trial on stage-IV colorectal cancer
Source: J Transl Med. 2016 Jan 12;14:10. doi: 10.1186/s12967-016-0765-4 (PMC4721000; doi:10.1186/s12967-016-0765-4)
Supplement: Supplementary file 1 — 10.1186/s12967-016-0765-4 Criteria for inclusion and exclusion of CRC patients used in the Oncogramme pilot trial. [file 12967_2016_765_MOESM1_ESM.pdf]

**Inclusion criteria**	**Exclusion criteria**
Age ≥ 18 years	Contraindication to paraclinic exploration necessary to patient follow-up
Stage-IV colorectal adenocarcinoma diagnosed	Use of concomitant chemotherapy, hormonotherapy or radiotherapy before final diagnosis
Surgical resection of primary tumor	Contraindication to chemotherapy
Treatment involving a standard chemotherapeutic protocol (5-FU + FA, FOLFIRI, FOLFOX)	Pregnancy, breastfeeding or absence of birthcontrol method for females of childbearing age
Tumor lesions must be measurable according to RECIST 2009	Difficulty to understand the whole clinical protocol
Absence of patient opposition	Absence of social security
-	Patients under legal protection

Table S1
Criteria for inclusion and exclusion of CRC patients used in the Oncogramme pilot trial.
