# Supplementary material for: Chemotherapy outcome predictive effectiveness by the Oncogramme: pilot trial on stage-IV colorectal cancer
Source: J Transl Med. 2016 Jan 12;14:10. doi: 10.1186/s12967-016-0765-4 (PMC4721000; doi:10.1186/s12967-016-0765-4)
Supplement: Supplementary file 2 — 10.1186/s12967-016-0765-4 Oncogramme profiles for 4 metastatic CRC patients included in the study. These profiles illustrate the heterogeneity of responses that occur from patient to patient, and for the three administered therapies. Bold dotted vertical line indicates on each graph the positivity threshold: an Oncogramme result indicative of resistance to the considered treatment is materialized by a red column extending to the left of threshold, an Oncogramme result indicative of sensitivity is materialized by a blue column extending to the right of threshold. [file 12967_2016_765_MOESM2_ESM.pdf]

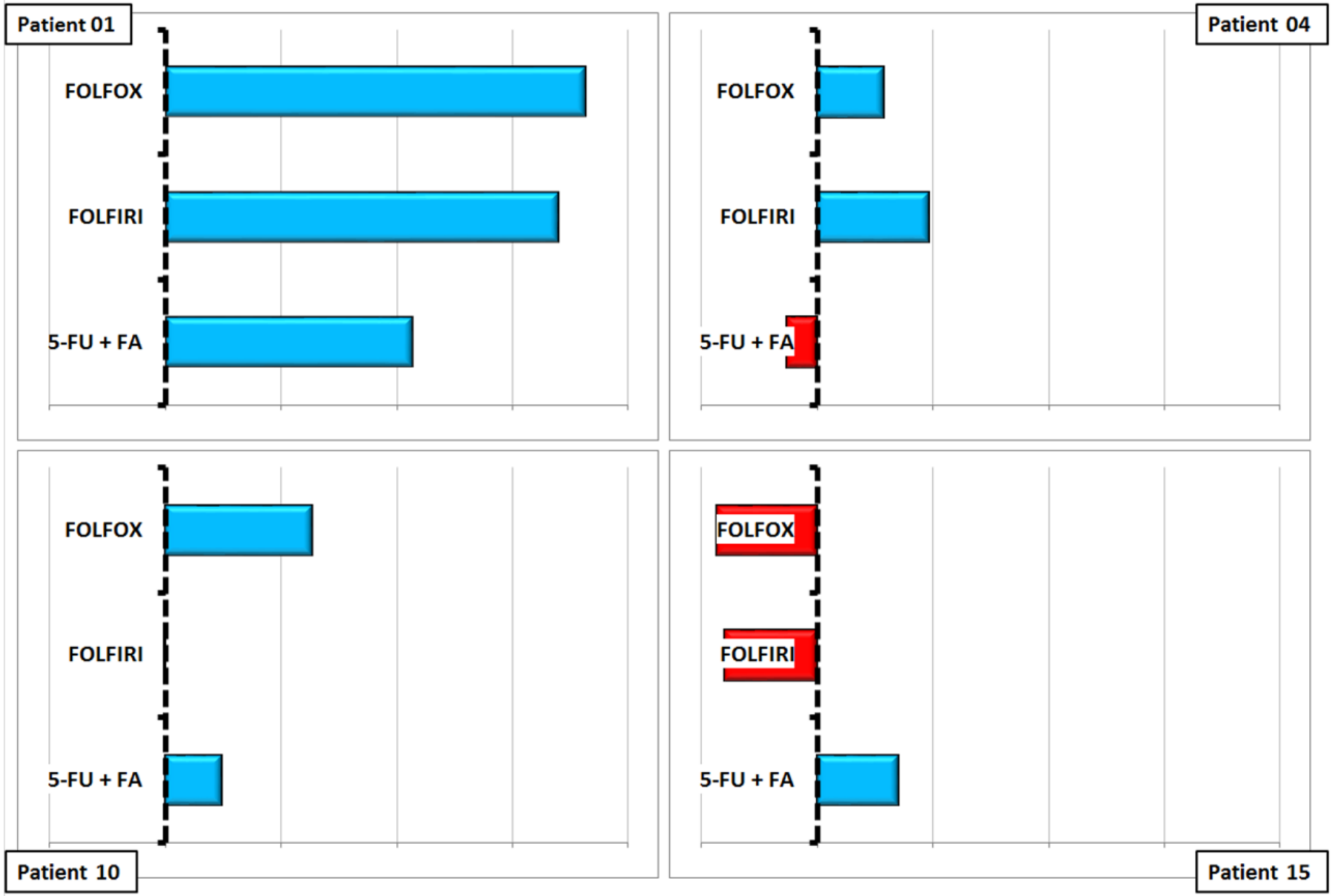

Figure S1

Oncogramme profiles for 4 metastatic CRC patients included in the study. These profiles illustrate the heterogeneity of responses that occur from patient to patient, and for the three administered therapies. Bold dotted vertical line indicates on each graph the positivity threshold: an Oncogramme result indicative of resistance to the considered treatment is materialized by a red column extending to the left of threshold, an Oncogramme result indicative of sensitivity is materialized by a blue column extending to the right of threshold.
